# A Comparative Study of Atropine, Orthokeratology, and Combination Therapy for Axial Elongation in Children with Myopia

**DOI:** 10.3390/jcm15187108

**Published:** 2026-09-13

**Authors:** Ji Ho Park, Chan-Ho Cho, Jaehan Joo, Moon Won Hwang, Dongwon Yi, Do Hee Jung, Jung Hyo Ahn, Seung Min Lee, Sangwoo Moon, Cheng-Ye Che, Su-Jin Kim, Ji Eun Lee

**Affiliations:** 1 Department of Ophthalmology, Pusan National University Yangsan Hospital, Pusan National University School of Medicine, Yangsan 50612, Republic of Korea; jihopark529@gmail.com (J.H.P.); jdh414@naver.com (D.H.J.); jhahn77@daum.net (J.H.A.); platinummetal@hanmail.net (S.M.L.); anstkddn0421@hanmail.net (S.M.); 2 Research Institute for Convergence of Biomedical Science and Technology, Pusan National University Yangsan Hospital, Yangsan 50612, Republic of Korea; 3 Department of Ophthalmology, College of Medicine, Yeungnam University, Daegu 42415, Republic of Korea; chanhocho@ynu.ac.kr; 4 Research Institute of Electronics and Information Communication, College of Information Technology and Convergence, Pukyong National University, Busan 48547, Republic of Korea; jhjoo@pknu.ac.kr; 5 Department of Ophthalmology, College of Medicine, Inje University Busan Paik Hospital, Inje University, Busan 48547, Republic of Korea; 6 Division of Endocrinology and Metabolism, Department of Internal Medicine, Pusan National University Yangsan Hospital, Pusan National University School of Medicine, Yangsan 50612, Republic of Korea; drwonny@pusan.ac.kr; 7 Department of Ophthalmology, Affiliated Hospital of Medical College, Qingdao University, Qingdao 266071, China; chechengye@126.com

**Keywords:** atropine, axial length, combination therapy, myopia control, orthokeratology

## Abstract

**Background/Objectives:** Atropine and orthokeratology (OK) are used for childhood myopia control, but comparisons of atropine monotherapy, OK monotherapy, and combination therapy remain limited. This study compared 12-month axial length changes and the proportions of fast, intermediate, and slow progressors among these treatments. **Methods:** This retrospective study included 154 children followed for at least 12 months: 70 received atropine monotherapy, 64 received OK monotherapy, and 20 received combination therapy. Axial length changes at 6 and 12 months were analyzed using a linear mixed-effects model adjusted for age at treatment initiation, sex, baseline axial length and baseline spherical equivalent refractive error. Based on 12-month axial elongation, progression was classified as fast (≥0.30 mm), intermediate (0.15 to <0.30 mm), or slow (<0.15 mm). **Results:** At 12 months, mean axial elongation was 0.294 ± 0.209 mm, 0.238 ± 0.228 mm, and 0.160 ± 0.109 mm in the atropine, OK, and combination groups, respectively. The group-by-time interaction was significant (*p* = 0.006). No significant pairwise differences were observed at 6 months. At 12 months, axial elongation was significantly smaller in the combination group than in the atropine and OK groups (adjusted mean differences, 0.161 and 0.115 mm; 95% CI, 0.078–0.245 and 0.024–0.207; Holm-adjusted *p* < 0.001 and 0.027, respectively). There was no significant difference between the atropine and OK groups (*p* = 0.155). The pattern of progression differed among groups (*p* = 0.017), with fast progressors least frequent in the combination group (5.0% vs. 32.9% and 32.8%). **Conclusions:** In this observational cohort, combination therapy was associated with less axial elongation and fewer fast progressors than either monotherapy.

## 1. Introduction

Myopia is one of the most common ocular disorders in children, and its prevalence is increasing rapidly worldwide, particularly in East Asia [1,2,3]. Earlier onset of myopia is associated with faster progression and a greater likelihood of developing high myopia later in life [2]. As myopia progression is driven largely by axial elongation, excessive ocular growth during childhood has emerged as a major clinical concern [4]. Progressive axial elongation is closely associated with sight-threatening complications such as retinal detachment, myopic macular degeneration, and glaucoma, underscoring the importance of controlling axial length growth during childhood [4,5].

Topical atropine is one of the most widely studied pharmacologic interventions for myopia control [6,7,8,9]. In the ATOM1 study, atropine 1% showed substantial efficacy in slowing myopia progression, but its clinical usefulness was limited by treatment-related adverse effects [6]. Subsequent studies, including ATOM2, suggested that lower concentrations were better tolerated and associated with less rebound after discontinuation, making low-dose atropine more practical for long-term clinical use [7,8,9]. However, lower or moderate concentrations do not completely suppress axial elongation in all children [7,9]. In the LAMP study, although 0.05%, 0.025%, and 0.01% atropine all reduced axial elongation compared with placebo, mean axial length still increased over 1 year in every treatment group, indicating that atropine monotherapy remains incomplete for some patients [10].

Orthokeratology (OK) is another established treatment strategy for childhood myopia control [11,12,13,14,15]. Overnight wear of reverse-geometry lenses temporarily reshapes the cornea, providing clear daytime vision while also slowing axial elongation compared with conventional optical correction [11,12,13]. Previous studies, including LORIC, long-term follow-up data from Hiraoka et al., and Santodomingo-Rubido et al., have consistently shown that OK can reduce axial elongation in myopic children [12,13,14,15]. Nevertheless, the treatment effect is not absolute, and some children continue to show clinically meaningful progression despite OK wear [13,14,15]. This incomplete response suggests that OK monotherapy may also be insufficient for a subset of patients [13,14,15].

Given that both atropine and OK are effective but individually imperfect approaches for myopia control, combination treatment has attracted increasing clinical interest [16,17,18,19]. Previous studies have suggested that adding low-dose atropine to OK may provide greater suppression of axial elongation than OK alone [16,17,18,19]. However, most of these studies primarily focused on the additional benefit of atropine in children already undergoing OK treatment, that is, comparisons between OK monotherapy and atropine-plus-OK combination therapy [16,17,18]. Kinoshita et al. reported reduced axial elongation with combined OK and 0.01% atropine compared with OK alone [16], and subsequent studies by Wan and Wen similarly evaluated the additive effect of atropine on an OK background [17,18]. Thus, the existing literature has largely addressed whether atropine enhances the effect of OK, rather than directly comparing the three major treatment strategies used in real-world practice [16,17,18].

Accordingly, the present study aimed to compare longitudinal changes in axial length over 12 months among children treated with atropine monotherapy, OK monotherapy, and combination therapy. In addition, we evaluated differences in progression patterns among the three groups. By simultaneously comparing these three treatment strategies, this study aimed to provide comparative evidence relevant to treatment selection for myopia control in children.

## 2. Materials and Methods

### 2.1. Ethics Statement

This study was approved by the Institutional Review Board (IRB) of Pusan National University Yangsan Hospital (approval number: 55-2026-085), and was conducted in accordance with the principles of the Declaration of Helsinki.

### 2.2. Study Design and Participants

**Figure 1:**
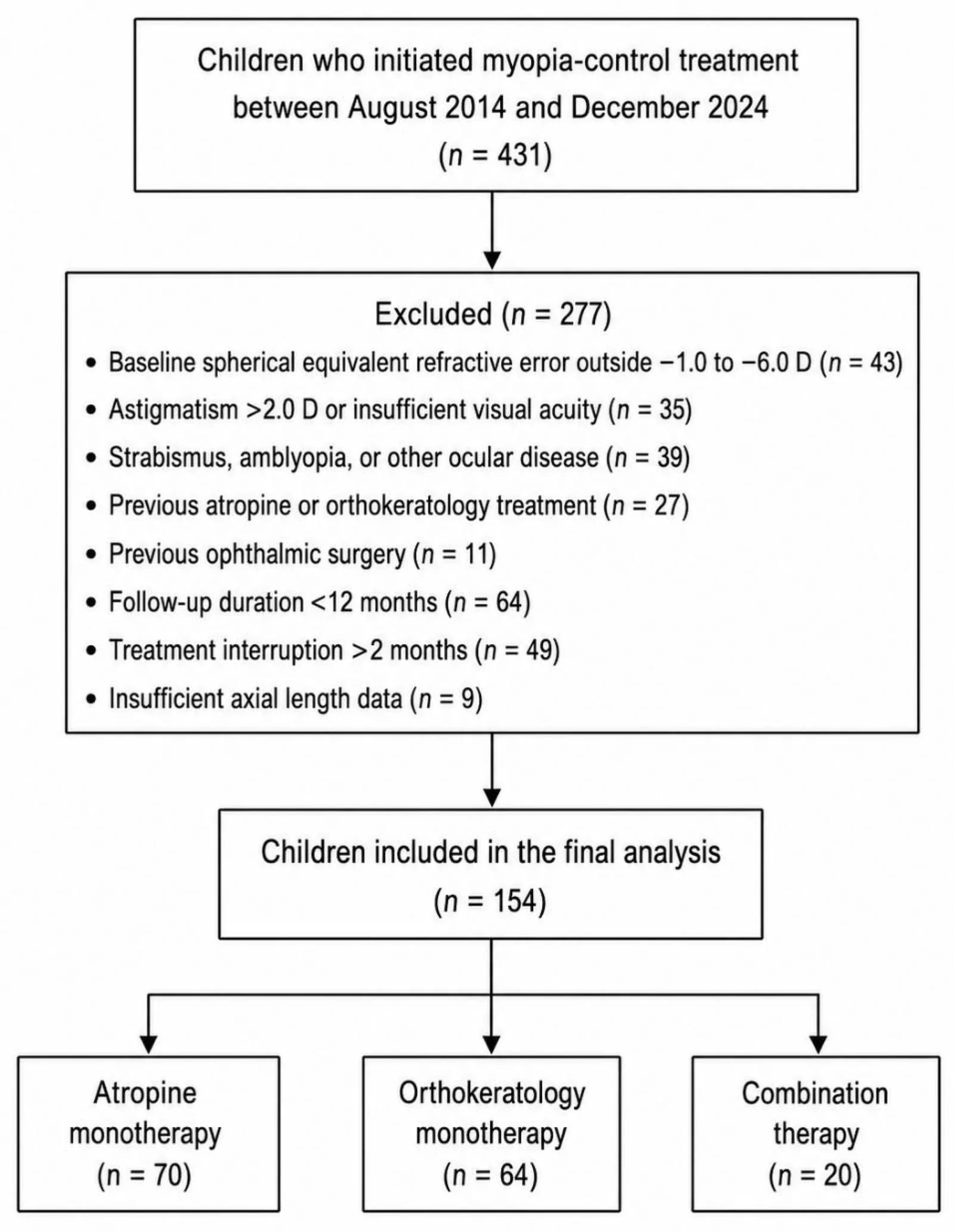
Flow diagram of participant selection.

This study was a retrospective review of medical records. Patients who initiated myopia-control treatment at Pusan National University Yangsan Hospital between 1 August 2014, and 31 December 2024 were screened for eligibility. Inclusion criteria were a cycloplegic spherical equivalent refractive error between −1.0 and −6.0 diopters (D) in both eyes, astigmatism of ≤2.0 D in both eyes, and best-corrected visual acuity (BCVA) of 0 logMAR or better. Patients were excluded if they had a history of ocular pathology other than refractive error, including strabismus, or amblyopia, had undergone previous ophthalmic surgery, or had previously used atropine or OK before. Patients were also excluded if treatment was interrupted for more than 2 months during the 12-month follow-up period or if the treatment duration was less than 12 months. For participants in whom both eyes met the eligibility criteria, one eye was randomly selected using a computer-generated random sequence before the outcome analysis. The participant selection process is summarized in Figure 1.

A total of 431 children who initiated myopia-control treatment between August 2014 and December 2024 were screened. After exclusion of 277 children who did not meet the eligibility criteria or had insufficient follow-up data, 154 children were included in the final analysis: 70 in the atropine monotherapy group, 64 in the orthokeratology monotherapy group, and 20 in the combination therapy group.

### 2.3. Treatment Protocol

Patients were classified into three groups according to the treatment they received. The treatment modality was selected after discussion between the treating physician and the parents regarding the available treatment options, expected benefits, potential risks, and practical considerations. Selection was based on the child’s age, refractive status, ability and willingness to wear contact lenses, parental preference, and the anticipated ability to adhere to treatment. The atropine monotherapy group used approximately 0.03% atropine ophthalmic solution, which was prepared by the hospital pharmacy by diluting commercially available 0.125% atropine sulfate ophthalmic solution (Myoguard^®^, LitePharmTech Co., Ltd., Seoul, Republic of Korea) with artificial tears at a ratio of 1:3. The prepared solution was dispensed to patients and instilled once daily. This formulation and dosing protocol was used throughout the study period. The OK monotherapy group wore orthokeratology lenses every night. Orthokeratology lenses used in this study included LK^®^ lenses (Lucid Korea Co., Ltd., Seoul, Republic of Korea), Emerald™ lenses (Euclid Systems Corporation, Dublin, OH, USA), and DMC FARGO-100 lenses (Dream Medical Company, Seoul, Republic of Korea). Among patients receiving orthokeratology, LK lenses were used in 53 of 64 patients (82.8%) in the orthokeratology monotherapy group and 16 of 20 patients (80.0%) in the combination group. Emerald lenses were used in 14 (21.9%) and 5 (25.0%) patients, respectively, while DMC FARGO-100 lenses were used in 6 (9.4%) patients in the OK monotherapy group and none in the combination group. Because some patients changed lens designs during follow-up, these categories were not mutually exclusive. If uncorrected distant visual acuity changed by more than 0.30 logMAR units during follow-up, the OK lenses were re-prescribed. The combination therapy group received both diluted atropine and nightly OK lens wear. All children in the combination group initiated atropine and orthokeratology concurrently; none were switched to combination therapy after documented failure of monotherapy. All patients were followed for at least 12 months.

### 2.4. Outcome Measures

The primary outcome was axial elongation from baseline at 6 and 12 months after treatment initiation. The secondary outcome was the distribution of slow, intermediate, and fast progressors at 12 months. Axial length was measured using the IOLMaster (Carl Zeiss Meditec AG, Jena, Germany). Patients were categorized according to 12-month axial length change as follows: slow progressors (<0.15 mm), intermediate progressors (0.15 to <0.3 mm), and fast progressors (≥0.3 mm). These study-defined categories were selected based on previous reports indicating that axial elongation of approximately 0.05–0.20 mm/year may represent physiological or low axial growth, whereas annual elongation of ≥0.30 mm has been considered indicative of relatively rapid progression or an inadequate treatment response [19,20]. Treatment-related adverse events documented in the medical records during follow-up were also retrospectively reviewed.

### 2.5. Statistical Analysis

**Figure 2:**
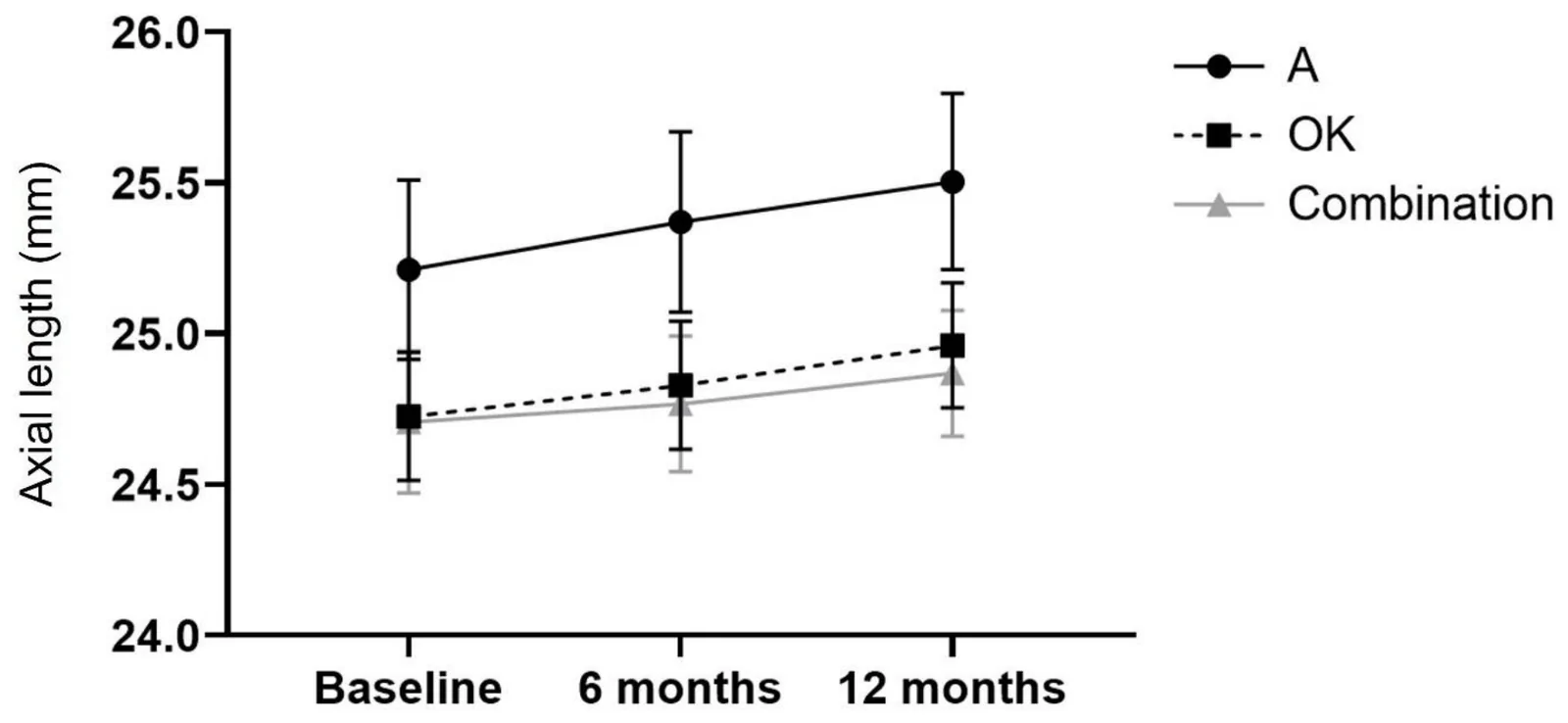
Longitudinal changes in axial length over 12 months. Mean axial length at baseline, 6 months, and 12 months is shown for participants with measurements available at all three time points. Complete-case sample sizes were 61, 63, and 20 in the atropine, orthokeratology, and combination groups, respectively. The same participants were represented at each visit within each treatment group. Axial length increased over time in all groups, with the combination therapy group showing the smallest observed increase. Error bars represent 95% confidence intervals. This figure presents complete-case descriptive data, whereas the descriptive summaries of observed axial elongation and the linear mixed-effects analysis used all available observations. A, Atropine; OK, Orthokeratology.

Continuous variables are presented as mean ± standard deviation, and categorical variables as number and percentage. Baseline characteristics were compared among the three groups using one-way analysis of variance (ANOVA) for continuous variables and the chi-square test for categorical variables. Axial elongation from baseline at 6 and 12 months was analyzed using a linear mixed-effects model with a subject-specific random intercept. Treatment group, follow-up time, and the group-by-time interaction were included as fixed effects, with adjustment for age at treatment initiation, sex, baseline axial length and baseline spherical equivalent refractive error. The model was estimated using restricted maximum likelihood. Pairwise comparisons among treatment groups were performed at each follow-up time point, with *p*-values adjusted using the Holm method. Model assumptions were assessed by visual inspection of quantile-quantile plots of the residuals and plots of residuals against fitted values. Because some departures from normality and homogeneity of residual variance were observed, a sensitivity analysis was performed after excluding participants with observations showing absolute standardized residuals greater than 3. An absolute standardized residual greater than 3 was used as a conventional criterion for identifying observations with extreme model residuals; participants meeting this criterion were excluded only in the sensitivity analysis. The mixed-effects model included all available follow-up measurements without requiring complete observations at both follow-up visits. No imputation was performed for missing 6-month measurements. For descriptive presentation, Figure 2 was based on participants with axial length measurements available at baseline, 6 months, and 12 months so that the plotted trajectories represented the same participants across all time points. In contrast, the descriptive summaries of observed axial elongation and the linear mixed-effects model used all available observations at each time point. A sensitivity analysis was additionally performed by including treatment initiation year as a continuous covariate in the linear mixed-effects model to account for potential temporal changes in myopia-control practice and treatment selection during the study period. The proportion of slow, intermediate, and fast progressors was compared using the chi-square test. To account for baseline differences among treatment groups, an additional multivariable analysis was performed with fast progression (12-month axial elongation ≥ 0.30 mm) as the dependent variable and treatment group, age at treatment initiation, sex, baseline axial length, and baseline spherical equivalent refractive error as independent variables. Because of the small number of fast progressors in the combination group, Firth penalized logistic regression was used to reduce small-sample bias and potential separation. A two-sided *p* < 0.05 was considered statistically significant. All statistical analyses were performed using IBM SPSS Statistics version 29 (IBM Corp., Armonk, NY, USA).

## 3. Results

A total of 154 children were included in the study: 70 in the atropine monotherapy group, 64 in the orthokeratology (OK) monotherapy group, and 20 in the combination therapy group. There were no significant differences among the three groups in age (*p* = 0.694), sex distribution (*p* = 0.717), spherical equivalent refractive error (*p* = 0.345), or uncorrected visual acuity (*p* = 0.247). However, baseline axial length differed significantly among the groups (*p* = 0.028) (Table 1).

The median interval from treatment initiation to the 6-month visit was 182 days (range, 172–195 days), and that to the 12-month visit was 364 days (range, 355–377 days). All 154 participants completed the 12-month follow-up. Axial length measurements at the 6-month visit were unavailable for 10 participants (9 in the atropine group and 1 in the OK group), although these participants attended the scheduled follow-up visit. Axial length measurements at 12 months were available for all participants.

Among participants with axial length measurements available at all three time points, axial length increased progressively over 12 months in all treatment groups (Figure 2). Based on all available observations at each follow-up visit, observed axial elongation at 6 months was 0.096 ± 0.114 mm in the atropine group, 0.106 ± 0.118 mm in the OK group, and 0.058 ± 0.044 mm in the combination group. At 12 months, observed axial elongation was 0.294 ± 0.209 mm in the atropine group, 0.238 ± 0.228 mm in the OK group, and 0.160 ± 0.109 mm in the combination group (Table 2). The smallest increase was observed in the combination therapy group, followed by the OK group, whereas the atropine group showed the greatest elongation.

**Figure 3:**
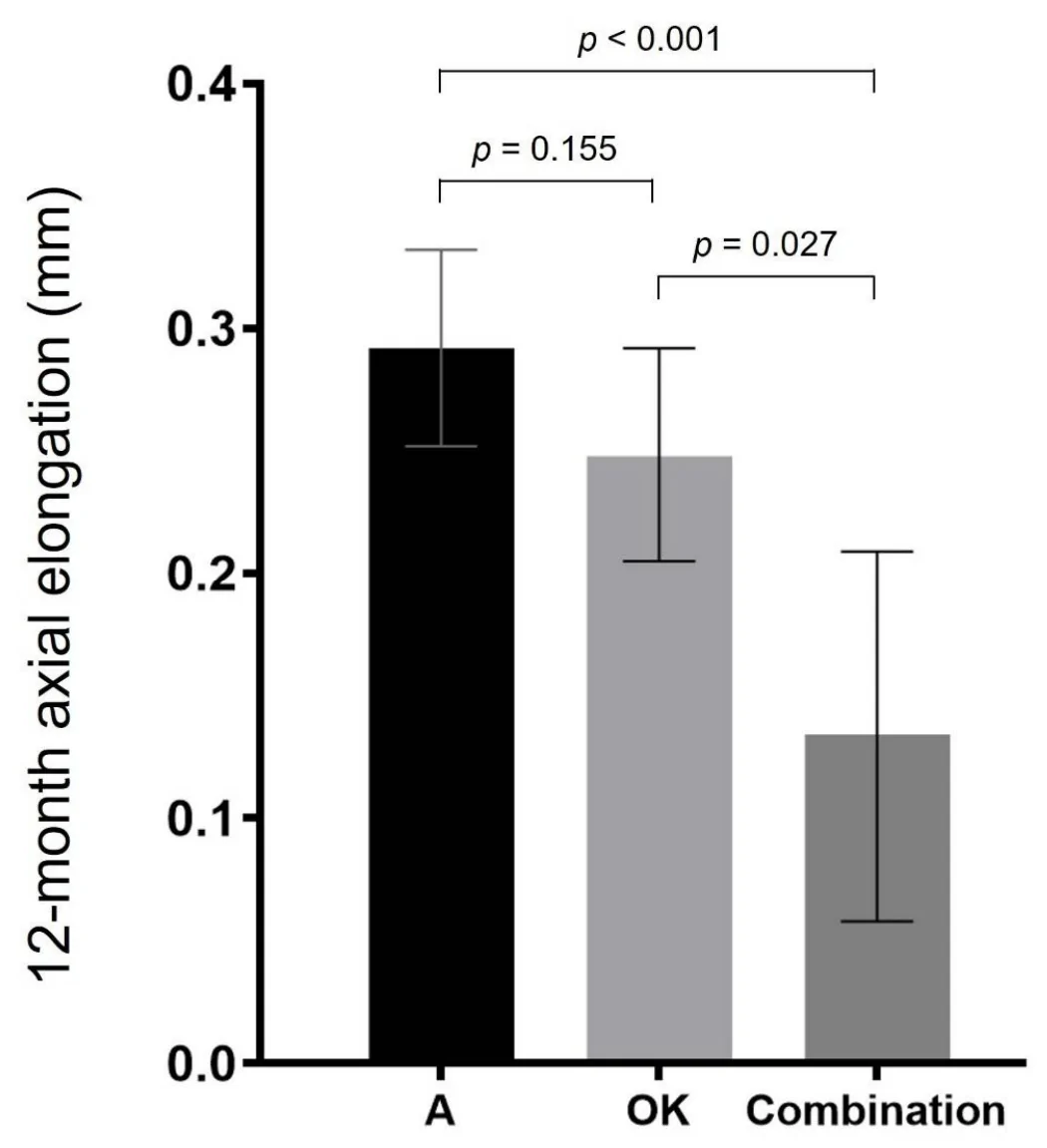
Estimated axial elongation at 12 months according to treatment group. Estimated changes in axial length from baseline to 12 months are presented for the atropine, orthokeratology, and combination therapy groups. Estimates were derived from a linear mixed-effects model including age at treatment initiation, sex, baseline axial length, baseline spherical equivalent refractive error, treatment group, follow-up time, and the group-by-time interaction, with a participant-specific random intercept. Error bars represent 95% confidence intervals. At 12 months, axial elongation was significantly smaller in the combination group than in the atropine group (Holm-adjusted *p* < 0.001) and the orthokeratology group (Holm-adjusted *p* = 0.027). No significant difference was observed between the atropine and orthokeratology groups (Holm-adjusted *p* = 0.155). A, Atropine; OK, Orthokeratology.

**Figure 4:**
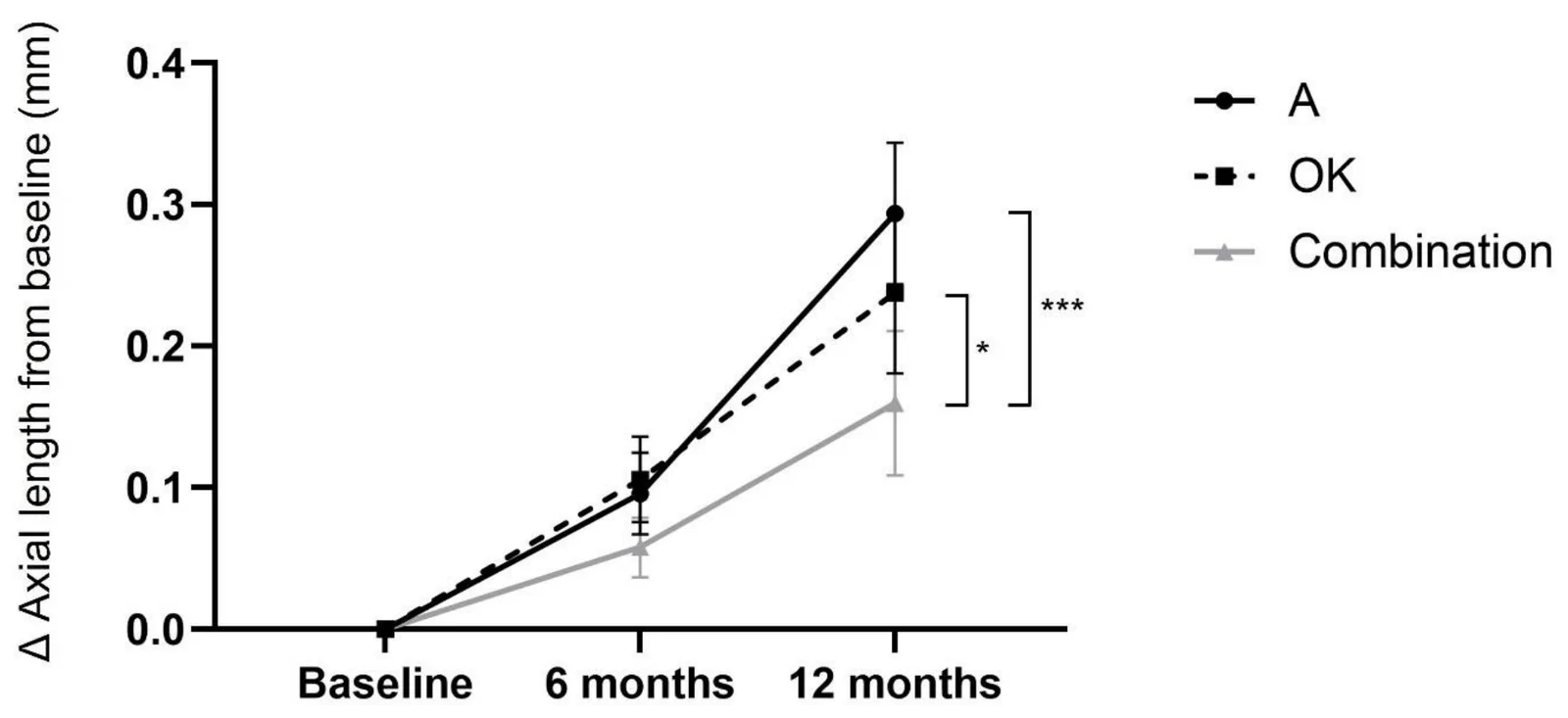
Changes in axial length from baseline over 12 months by treatment group. Points represent observed mean axial elongation, and error bars represent 95% confidence intervals. Lower values indicate less axial elongation. Between-group comparisons were performed using a linear mixed-effects model adjusted for age at treatment initiation, sex, baseline axial length and baseline spherical equivalent refractive error. No significant pairwise differences were observed at 6 months. At 12 months, axial elongation was significantly lower in the combination group than in the atropine and orthokeratology monotherapy groups. The group-by-time interaction was statistically significant (*p* = 0.006). A, Atropine; OK, Orthokeratology. * *p* < 0.05; *** *p* < 0.001.

In the linear mixed-effects model, the group-by-time interaction was statistically significant (*p* = 0.006), indicating that the pattern of axial elongation over time differed among the treatment groups. At 6 months, no significant pairwise differences were observed among the three groups. At 12 months, model-estimated axial elongation was smallest in the combination group and greatest in the atropine group (Figure 3). The model-estimated axial elongation at 12 months was 0.294 mm (95% CI, 0.253 to 0.334) in the atropine group, 0.247 mm (95% CI, 0.203 to 0.292) in the OK group, and 0.132 mm (95% CI, 0.057 to 0.208) in the combination group. Observed axial elongation over time showed a similar pattern, with no significant pairwise differences at 6 months and smaller axial elongation in the combination group than in either monotherapy group at 12 months (Figure 4). Axial elongation was significantly smaller in the combination group than in the atropine group (adjusted mean difference, 0.161 mm; 95% CI, 0.078 to 0.245; Holm-adjusted *p* < 0.001) and the OK group (adjusted mean difference, 0.115 mm; 95% CI, 0.024 to 0.207; Holm-adjusted *p* = 0.027). There was no significant difference between the atropine and OK groups (adjusted mean difference, 0.046 mm; 95% CI, −0.017 to 0.110; Holm-adjusted *p* = 0.155) (Table 3). Three participants had observations with absolute standardized residuals greater than 3. After excluding these participants, the group-by-time interaction remained statistically significant (*p* < 0.001), and the direction of the between-group differences was unchanged (Supplementary Table S1).

In a sensitivity analysis additionally adjusting for treatment initiation year, the overall group-by-time interaction remained significant (*p* = 0.006), and the estimated between-group differences were similar in magnitude to those of the primary model. However, the 12-month difference between the OK and combination groups no longer reached statistical significance after Holm adjustment (adjusted mean difference, 0.110 mm; 95% CI, 0.012 to 0.208; Holm-adjusted *p* = 0.055), whereas the difference between the atropine and combination groups remained significant (adjusted mean difference, 0.161 mm; 95% CI, 0.078 to 0.245; Holm-adjusted *p* < 0.001) (Supplementary Table S2).

**Figure 5:**
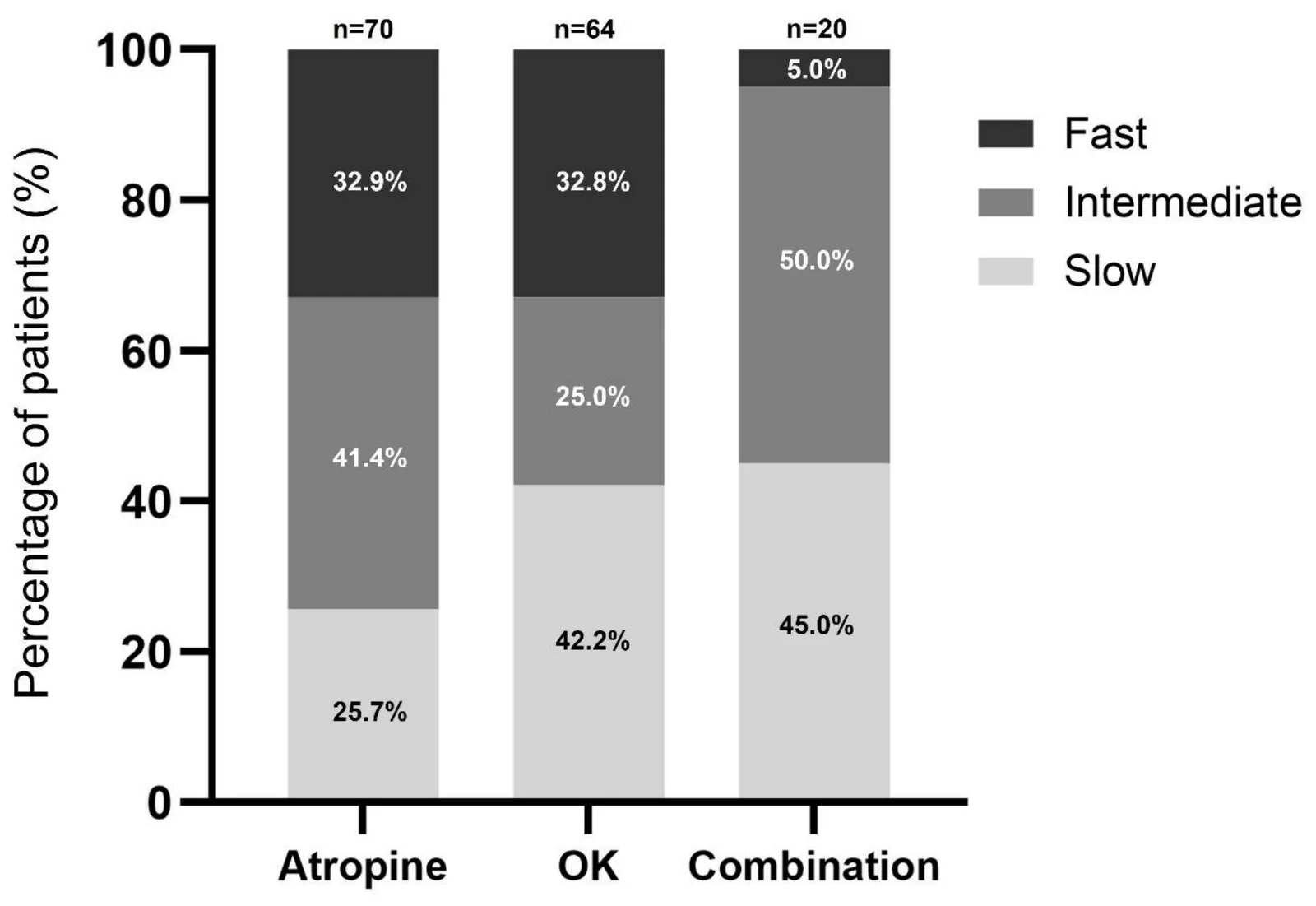
Distribution of slow, intermediate, and fast axial elongation at 12 months among the three treatment groups.

Patients were categorized into three groups according to 12-month axial length change: slow progressors (<0.15 mm), intermediate progressors (0.15 to <0.3 mm), and fast progressors (≥0.3 mm) (Figure 5). The proportion of slow progressors was highest in the combination group (9/20, 45.0%), followed by the OK group (27/64, 42.2%) and the atropine group (18/70, 25.7%). In contrast, the proportion of fast progressors was markedly lower in the combination group (1/20; 95% CI, 0.9–23.6%) compared with the atropine group (23/70; 95% CI, 23.0–44.5%) and the OK group (21/64; 95% CI, 22.6–45.0%). The combination group also showed the highest proportion of intermediate progressors (10/20, 50.0%), compared with the atropine group (29/70, 41.4%) and the OK group (16/64, 25.0%). Overall, the distribution of progression categories differed significantly among the three treatment groups (χ^2^ = 12.04, df = 4, *p* = 0.017). In the adjusted Firth logistic regression analysis, treatment group remained significantly associated with fast progression after adjustment for age at treatment initiation, sex, baseline axial length, and baseline spherical equivalent refractive error. Compared with atropine monotherapy, combination therapy was associated with lower odds of fast progression (adjusted OR, 0.065; 95% CI, 0.010–0.422; *p* = 0.004). Combination therapy was also associated with lower odds of fast progression than OK monotherapy (adjusted OR, 0.069; 95% CI, 0.010–0.478; *p* = 0.007), whereas no significant difference was observed between the atropine and OK monotherapy groups (adjusted OR, 0.950; 95% CI, 0.312–2.889; *p* = 0.928) (Supplementary Table S3).

Stacked bar graphs show the distribution of slow (<0.15 mm), intermediate (0.15 to <0.30 mm), and fast (≥0.30 mm) axial elongation in 12 months among the atropine monotherapy, orthokeratology (OK) monotherapy, and combination therapy groups. Group differences were analyzed using Pearson’s chi-square test (χ^2^ = 12.04, df = 4, *p* = 0.017).

Treatment-related adverse events were documented in 13 of 70 patients (18.6%) in the atropine group, 9 of 64 (14.1%) in the OK group, and 6 of 20 (30.0%) in the combination group. In the combination group, a total of 7 adverse events were documented in 6 patients, as 1 patient experienced 2 adverse events. The most commonly documented events were photophobia and ocular irritation or allergy in the atropine group, and ocular discomfort or pain and corneal epithelial abnormalities in the OK group. In the combination group, photophobia, ocular irritation or allergy, and ocular discomfort or pain were each documented in 2 of 20 patients (10.0%), and corneal staining or epithelial defects in 1 patient (5.0%). Temporary treatment interruption related to adverse events occurred in 2 (2.9%), 4 (6.3%), and 1 (5.0%) patients, respectively. No keratitis, corneal infection, or permanent treatment discontinuation due to adverse events was documented among patients included in the final analysis.

## 4. Discussion

To our knowledge, few studies have directly compared atropine monotherapy, orthokeratology monotherapy, and combination therapy within the same clinical cohort. In the present study, combination therapy was associated with the smallest axial elongation and the lowest observed proportion of fast progressors over 12 months. A linear mixed-effects model was used to account for repeated measurements within participants and the baseline differences among treatment groups. Baseline axial length differed among groups, with the atropine monotherapy group having a relatively longer baseline axial length compared with the other groups. This difference may reflect real-world treatment selection, in which treatment choice is influenced by degree of myopia, parental preference, and willingness to use contact lenses. After adjustment for age at treatment initiation, sex, baseline axial length, and baseline spherical equivalent refractive error, combination therapy was associated with less axial elongation over 12 months than either monotherapy, with adjusted between-group differences of 0.161 mm versus atropine and 0.115 mm versus orthokeratology. The overall consistency of the sensitivity analyses supports the robustness of the association between combination therapy and lower axial elongation, although the evidence for an additional benefit over orthokeratology alone was less conclusive. These findings should be interpreted as adjusted associations within this observational cohort rather than evidence of treatment superiority.

A notable finding of the present study is the absence of a statistically significant difference in axial elongation between atropine and orthokeratology monotherapy after multivariate adjustment. This observation is consistent with previous findings, with one-year axial elongation values for both treatments falling within comparable ranges across studies. In the LAMP study, 1-year axial elongation ranged from approximately 0.20 mm to 0.36 mm depending on atropine concentration [10]. Orthokeratology studies have reported 1-year axial elongation values that largely overlap with those observed in low-dose atropine studies, generally ranging from approximately 0.19 to 0.29 mm [13,14,15]. A recent meta-analysis similarly reported no significant difference in axial elongation between orthokeratology and low-dose atropine [21,22]. While certain studies have reported greater axial length suppression with orthokeratology than with low-dose atropine, especially in children with higher baseline myopia [21,23,24], the majority of evidence suggests that neither monotherapy is clearly superior, in line with our results.

Combination therapy demonstrated the smallest axial elongation in our cohort, with a mean increase of 0.160 mm over 12 months, and had the lowest proportion of fast progressors among the three groups. The extent of axial elongation observed in our combination group was broadly consistent with previous studies evaluating the additive effect of atropine in children undergoing orthokeratology. In a randomized clinical study by Kinoshita et al. [16], 1-year axial elongation was 0.09 mm in the combination group compared with 0.19 mm in the orthokeratology-only group, suggesting that the addition of atropine may enhance the treatment effect of orthokeratology. Similar findings have been reported by Wan et al. [17] and Wen et al. [18], and this overall pattern has also been supported by more recent meta-analytic evidence. A systematic review and meta-analysis by Queirós et al. [25] found that the addition of atropine to orthokeratology was associated with 0.09 mm less axial elongation than orthokeratology alone (95% CI, 0.06–0.12 mm). This meta-analysis showed a difference comparable to the adjusted 12-month difference of 0.115 mm observed between the orthokeratology and combination groups in our study. Notably, although atropine concentrations varied across studies ranging from 0.01% to 0.05%, with our study employing approximately 0.03%, the reported 1-year axial elongation values were generally within a similar range. Despite differences in atropine concentrations and study designs, the magnitude of axial elongation in our combination group was broadly comparable with that reported in previous studies. However, direct comparisons across studies should be interpreted cautiously because of differences in patient characteristics, treatment protocols, and study design.

The smaller axial elongation observed with combination therapy may be explained by the distinct and potentially complementary mechanisms of action of atropine and orthokeratology. Atropine is believed to act through pharmacologic pathways, specifically via muscarinic receptor blockade and modulation of scleral remodeling, whereas orthokeratology works through an optical mechanism related to corneal reshaping and peripheral myopic defocus [11,26]. Because the two treatments may influence ocular growth through partly distinct mechanisms, their combined use could plausibly provide additional suppression of axial elongation compared with either treatment alone. When administered as monotherapy, either treatment may be insufficiently effective in some children because of interindividual biological variability. In such cases, axial elongation may persist despite ongoing treatment, and these children may continue to be classified as ‘fast progressors’ even under myopia control therapy. By targeting two different pathways simultaneously, combination therapy may reduce this limitation of monotherapy and increase the likelihood of achieving better control of axial elongation, thereby contributing to the greater suppression of axial elongation observed in our cohort. However, the present observational study was not designed to investigate treatment mechanisms, and the observed clinical differences should not be interpreted as evidence of biological synergy.

This interpretation is further supported by our finding that the proportion of fast progressors was lowest in the combination group. This association persisted after adjustment for age at treatment initiation, sex, baseline axial length, and baseline spherical equivalent refractive error. In this context, the association with combination therapy may be reflected not only in lower mean axial elongation but also in a lower proportion of children meeting the fast-progressor criterion. This may be clinically relevant because comparisons based solely on mean axial elongation may not fully reflect differences in the proportion of children with rapid axial elongation. Previous studies on monotherapy have reported similar observations, noting that a subset of children continues to exhibit rapid axial elongation even when treated with low-concentration atropine or orthokeratology [27,28]. In particular, rapid axial elongation may persist in children receiving low-concentration atropine, especially among those who were younger at baseline, received 0.01% atropine, or had a shorter baseline axial length [27]. A similar pattern has been observed with orthokeratology, especially in children with younger treatment initiation, lower baseline myopia, or higher parental myopia [28]. Viewed in this context, our finding of a lower proportion of fast progressors in the combination group may provide additional clinically relevant information beyond comparisons of mean axial elongation alone.

Several limitations should be acknowledged. First, the retrospective design may have introduced selection bias, as treatment allocation was not randomized and could have been influenced by factors such as individual risk of myopia progression at baseline, parental preference, or willingness to use contact lenses. Although the analysis adjusted for age at treatment initiation, sex, baseline axial length, and baseline spherical equivalent refractive error, data on myopia progression and axial elongation before treatment initiation were not consistently available. Therefore, differences in pretreatment progression velocity among the groups could not be accounted for, and residual confounding from other unmeasured factors such as parental myopia, outdoor activity, near work, and treatment adherence cannot be excluded. In addition, because the study included only patients who maintained treatment for at least 12 months and excluded those with prolonged treatment interruptions, the study population may have preferentially represented patients with better treatment tolerance and adherence. Second, although significant between-group differences were observed in the primary analysis, the relatively small size of the combination group and the imbalance in sample size among the three groups limited the precision of the estimates and may have increased their sensitivity to individual observations. Therefore, the findings involving the combination group should be interpreted cautiously and confirmed in larger cohorts. Although some departures from normality and homogeneity of residual variance were observed, the main findings remained unchanged after exclusion of participants with large standardized residuals. Third, follow-up was limited to 12 months, precluding assessment of longer-term treatment effects, including the durability of benefit and potential rebound after treatment modification or discontinuation. In addition, the absence of an untreated or conventional spectacle-control group limited our ability to assess the absolute treatment effect of each strategy relative to the natural course of myopia progression. Larger real-world cohorts with balanced treatment groups, more comprehensive adjustment for treatment selection and adherence, and longer follow-up are needed to further define the comparative effectiveness of these strategies.

## 5. Conclusions

In conclusion, combination therapy with atropine and orthokeratology was associated with the smallest axial elongation and the lowest observed proportion of fast progressors over 12 months. These findings suggest that combination therapy may be associated with additional suppression of axial elongation compared with monotherapy. In addition, a key strength of this study is the direct comparison of atropine monotherapy, orthokeratology monotherapy, and combination therapy within a single cohort. While most previous studies have focused on adding atropine to ongoing orthokeratology, fewer have simultaneously evaluated all three treatment strategies. This design provides direct comparative evidence that may be relevant for clinical practice, where clinicians must often choose among these three approaches.

## Figures and Tables

**Table 1:** Baseline characteristics.

	Atropine Monotherapy (*n* = 70)	Orthokeratology Monotherapy (*n* = 64)	Combination Therapy (*n* = 20)	*p*-Value
Age (years)	9.26 ± 2.71	9.56 ± 1.93	9.40 ± 1.43	0.694
Gender (male:female)	29:41	31:33	9:11	0.717
Axial length (mm)	25.10 ± 1.14	24.67 ± 0.85	24.71 ± 0.50	0.028
Spherical equivalent refractive error (D)	−3.08 ± 1.13	−2.82 ± 1.08	−2.82 ± 1.07	0.345
Uncorrected visual acuity (logMAR)	0.63 ± 0.30	0.56 ± 0.27	0.60 ± 0.27	0.247

Data are presented as mean ± standard deviation. *p*-values were calculated using one-way analysis of variance (ANOVA) for continuous variables and Pearson’s chi-square test for categorical variables. A *p*-value < 0.05 was considered statistically significant. D, diopters.

**Table 2:** Observed axial length and axial elongation during follow-up.

	Baseline (mm)	6 Months (mm)	12 Months (mm)	Δ6 Months (mm)	Δ12 Months (mm)
Atropine monotherapy	25.101 ± 1.142 (*n* = 70)	25.306 ± 1.144 (*n* = 61)	25.395 ± 1.125 (*n* = 70)	0.096 ± 0.114 (*n* = 61)	0.294 ± 0.209 (*n* = 70)
Orthokeratology monotherapy	24.671 ± 0.850 (*n* = 64)	24.769 ± 0.852 (*n* = 63)	24.909 ± 0.832 (*n* = 64)	0.106 ± 0.118 (*n* = 63)	0.238 ± 0.228 (*n* = 64)
Combination therapy	24.706 ± 0.503 (*n* = 20)	24.764 ± 0.480 (*n* = 20)	24.866 ± 0.446 (*n* = 20)	0.058 ± 0.044 (*n* = 20)	0.160 ± 0.109 (*n* = 20)

Data are presented as observed mean ± standard deviation using all available observations at each time point. Δ6 months and Δ12 months represent changes from baseline among participants with measurements available at the corresponding follow-up visit. The number of available observations is shown in parentheses. Accordingly, the descriptive values in this table may differ slightly from those shown in Figure 2, which was restricted to participants with complete measurements at all three time points.

**Table 3:** Adjusted pairwise comparisons of axial elongation from the linear mixed-effects model.

Follow-Up	Comparison	Adjusted Mean Difference (mm)	95% CI	Holm-Adjusted *p*-Value
6 months	A-OK	−0.017	−0.082 to 0.048	0.608
	A-Combination	0.068	−0.016 to 0.152	0.224
	OK-Combination	0.085	−0.006 to 0.177	0.203
12 months	A-OK	0.046	−0.017 to 0.110	0.155
	A-Combination	0.161	0.078 to 0.245	<0.001
	OK-Combination	0.115	0.024 to 0.207	0.027

Adjusted mean differences were derived from a linear mixed-effects model including treatment group, follow-up time, and their interaction, adjusted for age at treatment initiation, sex, baseline axial length and baseline spherical equivalent refractive error. Positive values indicate greater axial elongation in the first-listed group. Pairwise *p*-values were adjusted using the Holm method within each follow-up time point. A, Atropine; OK, Orthokeratology; CI, confidence interval. Group-by-time interaction, *p* = 0.006.

## Data Availability

The data supporting the findings of this study are available from the corresponding author upon reasonable request.

## Supplementary Materials

The following supporting information can be downloaded at https://www.mdpi.com/article/10.3390/jcm15187108/s1, Table S1: Sensitivity analysis excluding participants with large standardized residuals; Table S2: Sensitivity analysis additionally adjusted for treatment initiation year; Table S3: Firth penalized logistic regression analysis for fast axial elongation at 12 months.

## Abbreviations

The following abbreviations are used in this manuscript:

ANOVA	Analysis of variance
BCVA	Best-corrected visual acuity
CI	Confidence interval
IRB	Institutional Review Board
logMAR	Logarithm of the minimum angle of resolution
OK	Orthokeratology
