# Supplementary material for: A Comparative Study of Atropine, Orthokeratology, and Combination Therapy for Axial Elongation in Children with Myopia
Source: J Clin Med. 2026 Sep 13;15(18):7108. doi: 10.3390/jcm15187108 (PMC13607194; doi:10.3390/jcm15187108)
Supplement: Supplementary file 1 [file jcm-15-07108-s001.zip › jcm-4511107-supplementary.pdf]

Table S1. Sensitivity analysis excluding participants with large standardized residuals.

Analysis	Participants	Group-by-time interaction
Primary analysis	154	$p = 0.006$
Sensitivity analysis	151	$p < 0.001$

The primary linear mixed-effects model included treatment group, follow-up time, and the group-by-time interaction as fixed effects, with adjustment for age at treatment initiation, sex, baseline axial length and baseline spherical equivalent refractive error. A subject-specific random intercept was included. The sensitivity analysis excluded three participants who had at least one observation with an absolute standardized residual greater than 3. The model structure and covariates were otherwise unchanged. *P*-values for the group-by-time interaction were obtained from joint Wald tests.

Table S2. Sensitivity analysis additionally adjusted for treatment initiation year.

12-month pairwise comparison	Primary model		Year-adjusted model	
	Adjusted difference (mm (95% CI))	Holm-adjusted *p*-value	Adjusted difference (mm (95% CI))	Holm-adjusted *p*-value
Atropine-OK	0.046 (-0.017 to 0.110)	0.155	0.051 (-0.022 to 0.124)	0.172
Atropine-Combination	0.161 (0.078 to 0.245)	**<0.001**	0.161 (0.078 to 0.245)	**<0.001**
OK-Combination	0.115 (0.024 to 0.207)	**0.027**	0.110 (0.012 to 0.208)	0.055

Adjusted differences represent model-based between-group differences in axial elongation at 12 months. Positive values indicate greater axial elongation in the first-listed group. The primary linear mixed-effects model included treatment group, time, the group-by-time interaction, age at treatment initiation, sex, baseline axial length, and baseline spherical equivalent, with participant-specific random intercepts. The sensitivity model additionally included treatment initiation year as a continuous covariate. Pairwise *p*-values were adjusted using the Holm method. CI, confidence interval.

Table S3. Firth penalized logistic regression analysis for fast axial elongation at 12 months.

Variable	Adjusted odds ratio	95% CI	*p*-value
			Overall *p*-value = 0.0002*
Atropine–OK	0.950	0.312-2.889	0.928
Atropine–Combination	0.065	0.010-0.422	0.004
OK–Combination	0.069	0.010-0.478	0.007
Age at treatment initiation, per year	0.840	0.736-0.959	0.010
Male sex	0.802	0.372-1.727	0.572
Baseline axial length, per mm	1.113	0.671-1.846	0.678
Baseline spherical equivalent, per D	1.181	0.978-1.427	0.084

Fast progression was defined as axial elongation ≥0.30 mm over 12 months. Firth penalized logistic regression was performed with fast progression as the dependent variable and treatment group, age at treatment initiation, sex, baseline axial length, and baseline spherical equivalent refractive error as independent variables. The analysis included 153 participants with complete covariate data. One participant in the orthokeratology group was excluded because baseline spherical equivalent refractive error was unavailable. Adjusted odds ratios are presented with 95% confidence intervals. *Overall *p*-value for treatment group based on the penalized likelihood-ratio test. CI, confidence interval; D, diopters.
